# Benefits of pancreatic parenchymal endoscopic ultrasonography in predicting microscopic precancerous lesions of pancreatic cancer

**DOI:** 10.1038/s41598-023-38920-1

**Published:** 2023-07-25

**Authors:** Kohei Yamakawa, Noriko Inomata, Atsuhiro Masuda, Mamoru Takenaka, Hirochika Toyama, Keitaro Sofue, Arata Sakai, Takashi Kobayashi, Takeshi Tanaka, Masahiro Tsujimae, Shigeto Ashina, Masanori Gonda, Shohei Abe, Shigeto Masuda, Hisahiro Uemura, Shinya Kohashi, Kae Nagao, Yoshiyuki Harada, Mika Miki, Yosuke Irie, Noriko Juri, Hideyuki Shiomi, Maki Kanzawa, Tomoo Itoh, Takumi Fukumoto, Yuzo Kodama

**Affiliations:** 1 Division of Gastroenterology, Department of Internal Medicine, Kobe University Graduate School of Medicine, 7-5-1 Kusunoki-Cho, Chuo-Ku, Kobe, Hyogo 650-0017 Japan; 2 Department of Gastroenterology and Hepatology, Kindai University Faculty of Medicine, Sayama, Japan; 3 Division of Hepato-Biliary-Pancreatic Surgery, Department of Surgery, Kobe University Graduate School of Medicine, Kobe, Japan; 4 Department of Radiology, Kobe University Graduate School of Medicine, Kobe, Japan; 5 Division of Gastroenterology and Hepato-Biliary-Pancreatology, Department of Internal Medicine, Hyogo Medical University, Nishinomiya, Japan; 6 Division of Diagnostic Pathology, Kobe University Graduate School of Medicine, Kobe, Japan

**Keywords:** Pancreatic disease, Ultrasonography, Cancer, Gastroenterology

## Abstract

Pancreatic cancer primarily arises from microscopic precancerous lesions, such as pancreatic intraepithelial neoplasia (PanIN) and acinar-to-ductal metaplasia (ADM). However, no established method exists for predicting pancreatic precancerous conditions. Endoscopic ultrasonography (EUS) can detect changes in pancreatic parenchymal histology, including fibrosis. This study aimed to elucidate the relationship between pancreatic parenchymal EUS findings and microscopic precancerous lesions. We retrospectively analyzed 114 patients with pancreatobiliary tumors resected between 2010 and 2020 and evaluated the association between pancreatic parenchymal EUS findings and the number of PanIN, ADM, and pancreatic duct gland (PDG). Of the 114 patients, 33 (29.0%), 55 (48.2%), and 26 (22.8%) had normal EUS findings, hyperechoic foci/stranding without lobularity, and hyperechoic foci/stranding with lobularity, respectively. Multivariate analyses revealed that abnormal EUS findings were significantly associated with the frequency of PanIN (hyperechoic foci/stranding without lobularity: OR [95% CI] = 2.7 [1.0–7.3], with lobularity: 6.5 [1.9–22.5], *P*_trend_ = 0.01) and ADM (hyperechoic foci/stranding without lobularity: 3.1 [1.1–8.2], with lobularity: 9.7 [2.6–36.3], *P*_trend_ = 0.003) but not with PDG (hyperechoic foci/stranding without lobularity: 2.2 [0.8–5.8], with lobularity: 3.2 [1.0–10.2], *P*_trend_ = 0.12). We observed a trend toward a significantly higher number of precancerous lesions in the following order: normal findings, hyperechoic foci/stranding without lobularity, and hyperechoic foci/stranding with lobularity. Pancreatic parenchymal EUS findings were associated with the increased frequency of PanIN and ADM. Lobularity may help predict the increased number of precancerous lesions.

## Introduction

Pancreatic ductal adenocarcinoma (PDAC) is solid cancer associated with a poor prognosis, with a 5-year survival rate of < 10%^1^. A major reason for this is that > 50% of the tumors are unresectable at the time of the diagnosis. In response, recent efforts have focused on risk stratification and screening high-risk patients for the early detection of PDAC^2^. Smoking, diabetes mellitus (DM), chronic pancreatitis (CP), and obesity are well-known risk factors for PDAC^3,4^. In addition, genetic syndromes or associated gene alterations have been identified as predisposing factors, such as hereditary pancreatitis (PRSS1), hereditary breast and ovarian cancer syndromes (BRCA1, BRCA2, PALB2), and Peutz–Jeghers syndrome (STK11)^3–5^. However, some PDAC cases may not present with these risk factors or genetic alterations, which limits the use of these factors alone for risk stratification. Therefore, novel approaches are required to more accurately assess the risk of developing PDAC to achieve earlier detection and improve patient outcomes.

PDAC mainly develops from two microscopic precancerous lesions: pancreatic intraepithelial neoplasia (PanIN) and intraductal papillary mucinous neoplasm (IPMN)^6,7^. Previous studies in mice have proposed that PanIN arises from acinar cells through acinar-to-ductal metaplasia (ADM)^8^, although it remains controversial in humans. Moreover, a recent study has reported that the pancreatic duct gland (PDG) is the compartment of origin for IPMN^9^, suggesting that PDG is a microscopic precancerous lesion. A progressive increase in PanIN incidence from the normal pancreas to CP and PDAC indirectly supports the precancerous role of PanINs in humans^10^, and an increased number of these precancerous pancreatic lesions in the background pancreas may correlate with the high risk of developing PDAC. Thus, the detection of these microscopic lesions can be used to predict the risk of developing PDAC.

Recently, there has been increasing interest in the use of imaging approaches for the early detection of PDAC. Imaging modalities such as endoscopic ultrasonography (EUS), magnetic resonance imaging, and computed tomography have shown promise in detecting early-stage PDAC and its precursor lesions^2^. Of these modalities, EUS, which has a high spatial resolution, can capture minor changes in the pancreas that other imaging modalities cannot^11^. However, even EUS has difficulty in directly detecting microscopic precancerous lesions without secondary findings such as pancreatic branch-duct dilatation. Abnormal EUS findings of the pancreatic parenchyma, such as hyperechoic foci/stranding and lobularity, have been identified as representative CP-EUS findings and are included in the diagnostic criteria of early CP in the Rosemont classification^12^ and Japanese diagnostic criteria 2019 (DC2019)^13^. Our previous studies and other studies have reported that these EUS findings of pancreatic parenchyma are associated with CP histological conditions, such as fibrosis, inflammation, and atrophy in patients with and without CP^14–17^. Given these findings, we hypothesized that the pancreatic parenchymal EUS findings could indirectly predict precancerous pancreatic lesions through the histological changes of the pancreas.

This study aimed to clarify whether the pancreatic parenchymal EUS findings could predict increased microscopic precancerous lesions, such as PanIN, ADM, and PDG. We retrospectively analyzed over 100 surgically resected specimens of pancreatobiliary diseases.

## Methods

### Patients and data collection

**Figure 1:**
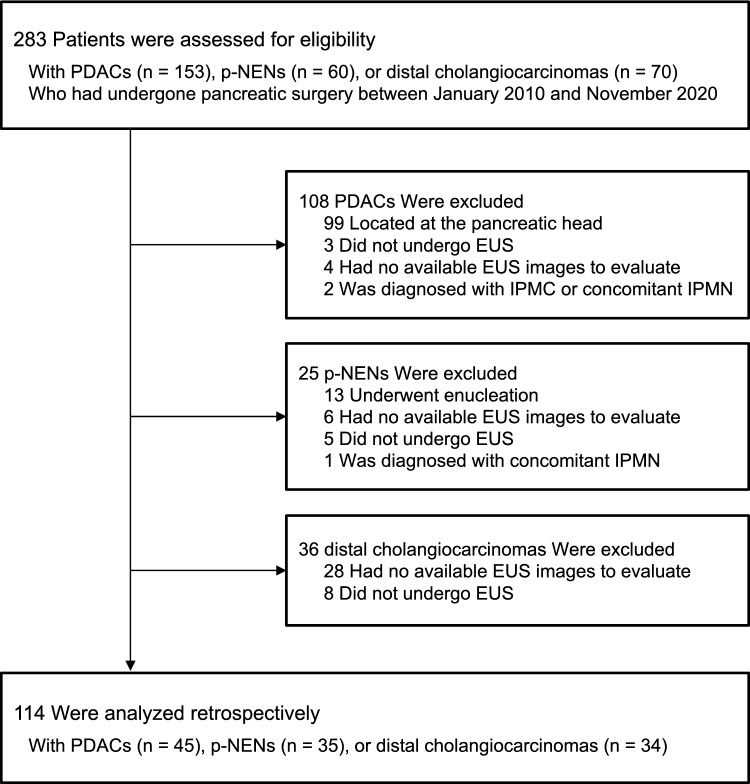
Study flowchart of patient inclusion criteria.

We retrospectively analyzed 114 consecutive patients with pancreatobiliary tumors who had undergone preoperative EUS and pancreatic surgery between January 2010 and November 2020 at the Kobe University Hospital (Fig. 1). Pancreatobiliary tumors included PDACs in the pancreatic body or tail, pancreatic-neuroendocrine neoplasms (p-NENs), and distal cholangiocarcinomas. This study excluded PDACs in the head of the pancreas because of their greater impact on the body and tail of the pancreas by occluding the main pancreatic duct (MPD). Moreover, as a result of the difficulty in distinguishing low-grade PanINs from IPMNs, we excluded patients with IPMNs. No patient met the definition of CP as per the DC2019 guidelines^13^. Clinical information included age, sex, body mass index (BMI), DM, alcohol consumption, smoking status, and the type of underlying tumor necessitating pancreatic surgery as in the same method reported previously^17^. The primary outcome of this study was the association between EUS findings and the frequency of microscopic precancerous lesions, including PanINs, ADMs, and PDGs. The secondary outcomes included the associations between EUS findings and histological findings and between histological findings and the frequency of microscopic precancerous lesions. This study was approved by the Kobe University Clinical Research Ethical Committee (approval number B210183) and performed in accordance with the Declaration of Helsinki. Informed consent was waived owing to the retrospective nature of the study, and the study information was disclosed on our hospital website, providing eligible patients the choice to opt-out.

### Evaluation of the pancreatic parenchymal EUS findings

We performed EUS using echo-endoscopes (GF-UCT240, GF-UCT260, or GF-UE260 [Olympus, Tokyo, Japan]) and EUS processors (ProSound α10, Aloka Arietta 850 [HITACHI, Tokyo, Japan] or EU-ME2 Premier Plus [Olympus, Tokyo, Japan]). We assessed the pancreatic parenchymal EUS findings such as hyperechoic foci/stranding and lobularity in the pancreatic body according to DC2019^13^ using a method reported previously^17^ (Supplementary Fig. S1a-c). Briefly, two experienced endosonographers (N.I. and A.M.), blinded to the clinical information of the patients, reviewed the EUS findings independently. A strong correlation was found between the two endosonographers in the evaluation of EUS findings of the pancreatic parenchyma (κ = 0.77 for hyperechoic foci/stranding, *P* < 0.001; κ = 0.77 for lobularity, *P* < 0.001). For cases diagnosed differently by these endosonographers, they examined the EUS images together to reconcile the diagnoses.

### Histological evaluation of precancerous pancreatic lesions

Histological findings were evaluated using a tissue section from the pancreatic resection margin, specifically at a site located at the pancreatic body or the nearest location to it, to match the EUS evaluation site as closely as possible. For patients with PDAC in the body/tail of the pancreas, histological findings were evaluated at the cranial side of the tumor to avoid the influences of obstructive pancreatitis on the pancreatic parenchyma. For p-NEN, histological findings were evaluated at the caudal and cranial sides of the tumor in the cases with pancreatic head and body/tail tumors, respectively, because no included patient with p-NEN had MPD occlusion. For patients with distal cholangiocarcinoma who underwent pancreaticoduodenectomy, histological findings were evaluated at the caudal side of the tumor. No patients with distal cholangiocarcinoma had MPD occlusion.

**Figure 2:**
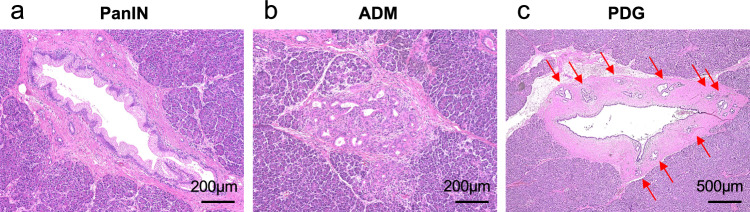
A representative histological picture of microscopic precancerous lesions. (**a**) Pancreatic intraepithelial neoplasia (PanIN). (**b**) Acinar-to-ductal metaplasia (ADM). (**c**) Pancreatic duct gland (PDG).

This study defined PanINs, ADMs, and PDGs as the microscopic precancerous lesions of PDAC and assessed them (Fig. 2a–c). Further, we assessed only low-grade PanINs because no patient had high-grade PanIN. Low-grade PanINs were defined as flat, micropapillary, or papillary noninvasive intraductal lesions that develop within small pancreatic ducts (< 5 mm)^18^. ADMs were defined as abnormally transformed lesions of mature acinar cells to cells with ductal differentiation^19^. PDGs were defined as normal gland-like outpouchings budding off pancreatic ducts^9^. PanIN, ADM, and PDG compartments were identified in each tissue section. The number (lesions) of PanIN, ADM, and PDG compartments was manually counted. The total number of lesions was divided by the total tissue area analyzed (cm^2^) to compare cases with different tissue section sizes, referring to a previous report^20^. This method standardized the frequency of microscopic precancerous lesions, expressed as lesions per square centimeter (lesions/cm^2^).

Further, we assessed histological conditions of the pancreatic parenchyma, such as fibrosis, inflammation, and atrophy (Supplementary Fig. S2a–l), using the same method reported previously^17^ and the same sections used to evaluate microscopic precancerous lesions. Briefly, two experienced pathologists (M.K. and T.I.) reviewed and evaluated the hematoxylin and eosin-stained tissue sections from all cases. The histological severity was classified into four grades: none, mild, moderate, or severe. A strong correlation was found between the two pathologists in the histological evaluation of the pancreatic parenchyma (κ = 0.95 for fibrosis, *P* < 0.001; κ = 0.94 for inflammation, *P* < 0.001; κ = 0.94 for atrophy, *P* < 0.001). In cases in which the two pathologists made different diagnoses, they examined the slides together to reconcile their findings. This study defined none of the histological grades as “absence” and mild, moderate, or severe histological grades as “presence.”

### Statistical analysis

All statistical analyses were conducted using GraphPad Prism 8 (GraphPad Software, La Jolla, CA) and IBM SPSS Statistics for Windows, Version 27.0 (IBM Corp., Armonk, NY, USA). A trend toward a higher number of microscopic precancerous lesions, such as PanINs, ADMs, and PDGs, was observed in the following ascending order: normal findings, hyperechoic foci/stranding without lobularity, and hyperechoic foci/stranding with lobularity. Thus, our primary hypothesis was tested using the linear trend test in an ordinal logistic regression model to assess the association between EUS findings (normal, hyperechoic foci/stranding without lobularity, or hyperechoic foci/stranding with lobularity as an ordinal variable), microscopic precancerous lesions (“high” or “low” frequency of PanINs, ADMs, or PDGs as a categorical variable), and histological features (absence or presence of pancreatic fibrosis, inflammation, or atrophy as a categorical variable). Those with a “high” frequency of microscopic precancerous lesions were defined as having a frequency (lesions/cm^2^) equal to or higher than the median frequency, whereas those with a “low” frequency were those with a frequency (lesions/cm^2^) less than the median number. The binary categorical variable (“high” or “low”) of microscopic precancerous lesions was used as an outcome variable. The multivariate model was adjusted for four covariates: age, smoking status (as a categorical variable: never, former, current), alcohol consumption, and the type of underlying tumor necessitating pancreatic surgery. A backward stepwise elimination with a threshold of *P* = 0.05 was used to select covariates in the final models. The chi-square test (or Fisher’s exact test, if appropriate) was used for the statistical comparison of categorical data. For the statistical comparison of continuous data, a two-tailed t-test and an analysis of variance followed by Tukey’s test were used. Statistical significance was set at *P* < 0.05.

## Results

### Patient characteristics with each pancreatic parenchymal EUS finding

First, based on EUS findings in the pancreatic parenchyma, we classified the 114 patients into normal, hyperechoic foci/stranding without lobularity, and hyperechoic foci/stranding with lobularity (Table 1). Of the 114 patients, 33 (29.0%) had normal EUS findings, 55 (48.2%) had hyperechoic foci/stranding without lobularity, and 26 (22.8%) had hyperechoic foci/stranding with lobularity. All patients with lobularity had hyperechoic foci/stranding.

**Table 1:** Classification of the 114 included patients based on endoscopic ultrasonography findings in the pancreatic parenchyma.

		Hyperechoic foci/stranding	
		Absence	Presence
Lobularity	Absence	Normal	Hyperechoic foci/stranding without lobularity
		33 (29.0%)	55 (48.2%)
	Presence	0 (0.0%)	Hyperechoic foci/stranding with lobularity
			26 (22.8%)

The data are expressed as numbers (percentage).

We then compared the patient characteristics with each EUS finding in the pancreatic parenchyma (Table 2). Regarding the smoking status, the proportion of current smokers tended to be high among those with hyperechoic foci/stranding with and without lobularity than among those with normal findings, although there was no significant difference (*P* = 0.06). Other than the smoking status, no significant difference was found in the following clinical characteristics: age, sex, BMI, alcohol consumption, DM, and the underlying tumor necessitating pancreatic surgery.

**Table 2:** Patient characteristics with each pancreatic parenchymal endoscopic ultrasonography finding.

		Normal	Hyperechoic foci/stranding		*P*-value
			Without lobularity	With lobularity	
All patients	114	33	55	26	
Mean age ± SD (years)	67.0 ± 12.7	63.8 ± 14.8	69.2 ± 10.6	66.4 ± 13.6	0.16
Sex					0.87
Men	64 (54.7%)	19 (57.6%)	29 (52.7%)	15 (57.7%)	
Women	53 (45.3%)	14 (42.4%)	26 (47.3%)	11 (42.3%)	
BMI ± SD (kg/m^2^)	21.2 ± 2.7	20.9 ± 2.4	21.4 ± 3.0	21.3 ± 2.6	0.72
Smoking status					0.060
Never	66 (57.9%)	21 (63.6%)	32 (58.2%)	13 (50.0%)	
Former	37 (32.5%)	12 (36.4%)	17 (30.9%)	8 (30.8%)	
Current	11 (9.6%)	0 (0.0%)	6 (10.9%)	5 (19.2%)	
Alcohol consumption					0.47
≥ 60 g/day	20 (17.5%)	5 (15.2%)	12 (21.8%)	3 (11.5%)	
< 60 g/day	94 (82.5%)	28 (84.8%)	43 (78.2%)	23 (88.5%)	
DM					0.73
Presence	26 (22.8%)	6 (18.2%)	14 (25.5%)	6 (23.1%)	
Absence	88 (77.2%)	27 (81.8%)	41 (74.5%)	20 (76.9%)	
Underlying tumor necessitating pancreatic surgery					0.071
PDAC	45 (39.5%)	12 (36.3%)	26 (47.3%)	7 (26.9%)	
p-NEN	35 (30.7%)	15 (45.5%)	12 (21.8%)	8 (30.8%)	
Distal cholangiocarcinoma	34 (29.8%)	6 (18.2%)	17 (30.9%)	11 (42.3%)	

The data are expressed as numbers (percentage) and mean ± SD. Percentage (%) indicates the proportion of cases with specific clinical features in patients with each endoscopic ultrasonography finding in the pancreatic parenchyma. *SD* standard deviation, *BMI* body mass index, *DM* diabetes mellitus, *PDAC* pancreatic ductal carcinoma, *p-NEN* pancreatic-neuroendocrine neoplasm.

### Frequency of occurrence of microscopic precancerous lesions in the pancreatic parenchyma in patients with each type of pancreatobiliary tumor

**Figure 3:**
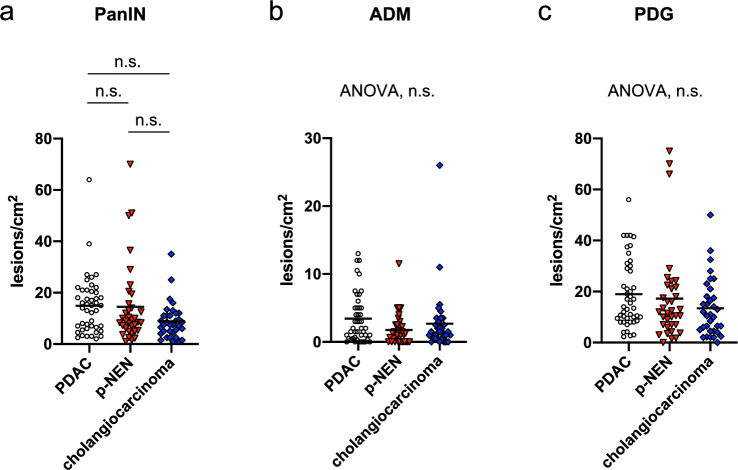
The frequency of occurrence of microscopic pancreatic lesions, such as pancreatic intraepithelial neoplasia (**a**), acinar-to-ductal metaplasia (**b**), and pancreatic duct gland (**c)**, in each underlying tumor necessitating pancreatic surgery. n.s., not significant; by one-way analysis of variance (ANOVA) with Tukey’s test in (**a**) or one-way ANOVA in (**b**) and (**c**).

To explore the influence of underlying tumors on the development of microscopic precancerous lesions in the background pancreas, we compared the frequency of occurrence of microscopic precancerous lesions in patients with each type of pancreatobiliary tumor (Fig. 3).

There was no significant difference in the frequency of PanIN, ADM, and PDG between the different types of underlying tumors.

### Each pancreatic parenchymal EUS finding associated with histological conditions

To confirm that pancreatic parenchymal EUS findings reflected histological conditions in this study’s population, we examined the association between parenchymal EUS findings and histological conditions, such as fibrosis, inflammation, and atrophy (Supplementary Table S1).

Lobularity, hyperechoic foci/stranding without lobularity, and normal findings were strongly associated with fibrosis in that order (hyperechoic foci/stranding without lobularity: multivariable odds ratio [OR] = 11.0, 95% confidence interval [CI] = 3.6–33.3; lobularity: multivariable OR = 65.7, 95% CI = 10.6–406.8; *P*_trend_ < 0.001), inflammation (hyperechoic foci/stranding without lobularity: multivariable OR = 20.3, 95% CI = 2.5–165.8; lobularity: multivariable OR = 112.6, 95% CI = 11.5–1103.2; *P*_trend_ < 0.001), and atrophy (hyperechoic foci/stranding without lobularity: multivariable OR = 7.6, 95% CI = 2.4–23.9; lobularity: multivariable OR = 59.6, 95% CI = 10.2–348.6; *P*_trend_ < 0.001). These results were consistent with our previous report^17^.

We further investigated the relationship between the surgical margin distance and EUS findings, the tumor size and EUS findings, the surgical margin distance and pathological findings, and the tumor size and pathological findings. Our analyses did not uncover any significant associations between these variables (Supplementary Fig. S3 and S4).

### Histological conditions of the pancreatic parenchyma were associated with the frequency of occurrence of microscopic precancerous lesions

Previous research indicated that microscopic precancerous lesions, including PanINs and ADMs, increased in response to chronic inflammation^7,21^. To confirm whether microscopic precancerous lesions increased as histological grades progressed, we investigated the association between histological conditions, such as fibrosis, inflammation, and atrophy, and the frequency of occurrence of each microscopic precancerous lesion in the pancreatic parenchyma.

Regarding fibrosis (Table 3), the frequency of occurrence of microscopic precancerous lesions, such as PanINs, ADMs, and PDGs, was significantly higher in the pancreas with fibrosis than in that without fibrosis (PanIN: multivariable OR = 3.1, 95% CI = 1.3–7.5, *P*-value = 0.01; ADM: multivariable OR = 3.6, 95% CI = 1.5–8.4, *P*-value = 0.004; PDG: multivariable OR = 2.7, 95% CI = 1.2–6.3,* P*-value = 0.021).

**Table 3:** Association of histological conditions of the pancreas with the frequency of occurrence of microscopic precancerous lesions.

Fibrosis	No. of cases with “high” frequency of PanIN	PanIN mean ± SD (lesion/cm^2^)	PanIN (outcome variable)	
			Univariate OR (95% CI)	Multivariate OR^**#**^ (95% CI)
Absence (none)	15 (34.1%)	8.5 ± 7.3	1 (reference)	1 (reference)
Presence (mild/moderate/severe)	41 (58.6%)	15.7 ± 13.5	2.7 (1.2–6.0)	3.1 (1.3–7.5)
*P*-value			0.01	0.01

Fibrosis	No. of cases with “high” frequency of ADM	ADM mean ± SD (lesion/cm^2^)	ADM (outcome variable)	
			Univariate OR (95% CI)	Multivariate OR^#^ (95% CI)
Absence (none)	15 (34.1%)	2.2 ± 4.6	1 (reference)	1 (reference)
Presence (mild/moderate/severe)	46 (65.7%)	3.0 ± 3.0	3.7 (1.7–8.2)	3.6 (1.5–8.4)
*P*-value			< 0.001	0.004

Fibrosis	No. of cases with “high” frequency of PDG	PDG mean ± SD (lesion/cm^2^)	PDG (outcome variable)	
			Univariate OR (95% CI)	Multivariate OR^#^ (95% CI)
Absence (none)	15 (34.1%)	11.1 ± 10.4	1 (reference)	1 (reference)
Presence (mild/moderate/severe)	41 (58.6%)	20.3 ± 15.7	2.7 (1.2–6.0)	2.7 (1.2–6.3)
*P*-value			0.01	0.02

^#^The odds ratio (OR) was adjusted for age, smoking status, alcohol consumption, and underlying tumor necessitating pancreatic surgery. *EUS* endoscopic ultrasonography, *SD* standard deviation, *PanIN* pancreatic intraepithelial neoplasia, *ADM* acinar-to-ductal metaplasia, *PDG* pancreatic duct gland, *OR* odds ratio, *CI* confidence interval.

Regarding inflammation (Supplementary Table S2) and atrophy (Supplementary Table S3), the frequency of occurrence of microscopic pancreatic precancerous lesions, such as PanINs, ADMs, and PDGs, was significantly more associated with inflammation and atrophy.

These results confirmed that all microscopic precancerous lesions increased with the progression in histological conditions of the pancreatic parenchyma, although PDGs were more weakly associated with histological conditions than PanINs and ADMs.

### Each pancreatic parenchymal EUS finding was associated with the frequency of occurrence of microscopic precancerous lesions

**Figure 4:**
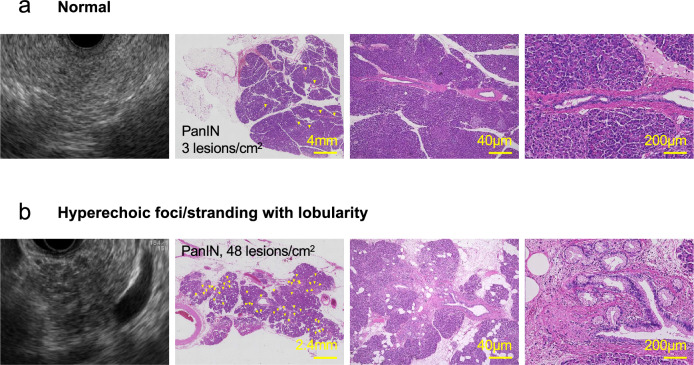
Comparison between EUS images and histology in representative cases. (**a**) A case with normal EUS findings. (**b**) A case with hyperechoic foci/stranding with lobularity. *Arrowhead* indicates the presence of PanINs.

We further evaluated the association between the frequency of occurrence of each microscopic precancerous lesion and each EUS finding in the pancreatic parenchyma (Fig. 4 and Supplementary Fig. S5). Our results suggested that normal findings, hyperechoic foci/stranding without lobularity, and hyperechoic foci/stranding with lobularity may have an ordinal correlation with the frequency of occurrence of each microscopic precancerous lesion (PanIN: normal 7.0 ± 4.8 vs. hyperechoic foci/stranding without lobularity 13.2 ± 8.9 vs. lobularity 20.2 ± 18.6 lesions/cm^2^; ADM: normal 1.4 ± 1.9 vs. hyperechoic foci/stranding without lobularity 2.8 ± 3.2 vs. lobularity 4.1 ± 5.5 lesions/cm^2^; PDG: normal 10.9 ± 8.4 vs. hyperechoic foci/stranding without lobularity 17.9 ± 14.6 vs. lobularity 21.8 ± 18.2 lesions/cm^2^).

We performed an ordinal logistic regression analysis to clarify whether each EUS finding correlated with the frequency of occurrence of microscopic precancerous lesions (Table 4). Our analyses revealed that lobularity, hyperechoic foci/stranding without lobularity, and normal findings strongly correlated with the frequency of PanINs in that order (hyperechoic foci/stranding without lobularity: multivariable OR = 2.7, 95% CI = 1.0–7.3; lobularity: multivariable OR = 6.5, 95% CI = 1.9–22.5; *P*_trend_ = 0.011) and ADMs (hyperechoic foci/stranding without lobularity: multivariable OR = 3.1, 95% CI = 1.1–8.2; lobularity: multivariable OR = 9.7, 95% CI = 2.6–36.3; *P*_trend_ = 0.003). Conversely, EUS findings did not significantly correlate with the frequency of PDGs (hyperechoic foci/stranding without lobularity: multivariable OR = 2.2, 95% CI = 0.8–5.8; lobularity: multivariable OR = 3.2, 95% CI = 1.0–10.2; *P*_trend_ = 0.12).

**Table 4:** Correlation of each pancreatic parenchymal endoscopic ultrasonography finding with the frequency of occurrence of microscopic precancerous lesions.

EUS finding	No. of cases with “high” frequency of PanIN	PanIN mean ± SD (lesion/cm^2^)	PanIN (outcome variable)	
			Univariate OR (95% CI)	Multivariate OR^**#**^ (95% CI)
Normal	10 (30.3%)	7.0 ± 4.8	1 (reference)	1 (reference)
Hyperechoic foci/stranding Without lobularity	28 (50.9%)	13.2 ± 8.9	2.4 (1.0–5.9)	2.7 (1.0–7.3)
With lobularity	18 (69.2%)	20.2 ± 18.6	5.2 (1.7–15.8)	6.5 (1.9–22.5)
*P*_trend_*			0.01	0.01

	No. of cases with “high” frequency of ADM	ADM mean ± SD (lesion/cm^2^)	ADM (outcome variable)	
			Univariate OR (95% CI)	Multivariate OR^**#**^ (95% CI)
Normal	10 (30.3%)	1.4 ± 1.9	1 (reference)	1 (reference)
Hyperechoic foci/stranding Without lobularity	32 (58.2%)	2.8 ± 3.2	3.2 (1.3–8.0)	3.1 (1.1–8.2)
With lobularity	19 (73.1%)	4.1 ± 5.5	6.2 (2.0–19.5)	9.7 (2.6–36.3)
*P*_trend_*			0.003	0.003

	No. of cases with “high” frequency of PDG	PDG mean ± SD (lesion/cm^2^)	PDG (outcome variable)	
			Univariate OR (95% CI)	Multivariate OR^**#**^ (95% CI)
Normal	11 (33.3%)	10.9 ± 8.4	1 (reference)	1 (reference)
Hyperechoic foci/stranding Without lobularity	29 (52.7%)	17.9 ± 14.6	2.2 (0.9–5.5)	2.2 (0.8–5.8)
With lobularity	16 (61.5%)	21.8 ± 18.2	3.2 (1.1–9.3)	3.2 (1.0–10.2)
*P*_trend_*			0.081	0.12

*P_trend_ was calculated by ordinal logistic regression analysis across the ordinal categories (normal, hyperechoic without lobularity, and lobularity) of EUS finding in the pancreatic parenchyma. # The odds ratio (OR) was adjusted for age, smoking status, alcohol consumption, and underlying tumor necessitating pancreatic surgery. *EUS* endoscopic ultrasonography, *SD* standard deviation, *PanIN* pancreatic intraepithelial neoplasia, *ADM* acinar-to-ductal metaplasia, *PDG* pancreatic duct gland, *OR* odds ratio, *CI* confidence interval.

## Discussion

**Figure 5:**
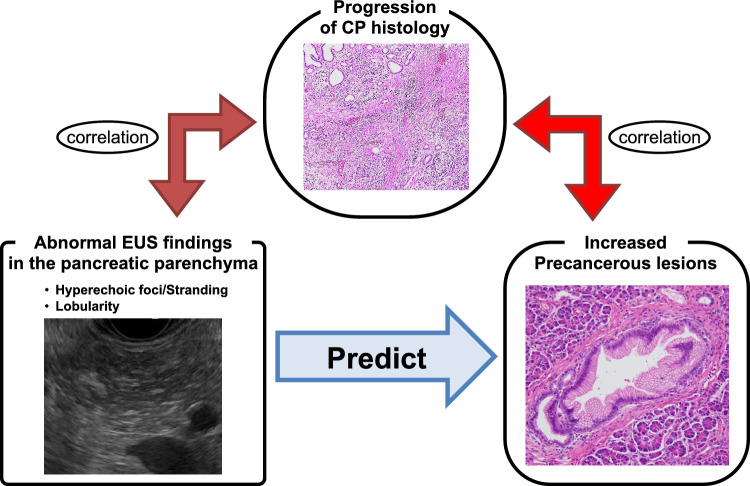
A schematic representation of the association between endoscopic ultrasonography findings, histological conditions, and microscopic precancerous lesions in the pancreatic parenchyma.

The current study revealed an association between EUS findings and the frequency of occurrence of microscopic precancerous lesions, especially PanINs and ADMs, in pancreatic parenchyma. Additionally, a good correlation was found between EUS findings and histological conditions and between histological conditions and the frequency of occurrence of microscopic precancerous lesions in the pancreas. Given that EUS cannot directly detect microscopic lesions, these results suggest that pancreatic parenchymal EUS findings may help indirectly predict an increased number of microscopic precancerous lesions through capturing macroscopic changes of histological conditions (Fig. 5). Furthermore, a trend toward a significantly higher number of PanINs and ADMs was observed in the following order: normal EUS findings, hyperechoic foci/stranding without lobularity, and hyperechoic foci/stranding with lobularity. We previously reported that CP histological grades significantly correlated with these EUS findings in a similar order^17^. The ordinality of the correlations supported our abovementioned conclusion. Several studies have reported that pancreatic parenchymal EUS findings correlate with fibrosis^14–16^ and that chronic inflammation, such as CP, is closely associated with increased microscopic precancerous lesions in humans and mice^21–23^. However, the literature on the direct relationship between pancreatic parenchymal EUS findings and microscopic precancerous lesions is limited. The novelty of this study lies in its specific aim to elucidate the predictive potential of pancreatic parenchymal EUS findings for microscopic precancerous lesions, especially PanINs and ADMs. Additionally, our results suggest that hyperechoic foci/stranding and lobularity are helpful as secondary findings of increased microscopic precancerous lesions.

In addition, we demonstrated that abnormal parenchymal EUS findings could predict an increased number of precancerous lesions, such as PanINs and ADMs, in patients without CP. According to a previous study, approximately 17% of patients who underwent EUS not for pancreatobiliary disease showed abnormal findings in the pancreatic parenchyma [24]. We reported that abnormal parenchymal EUS findings were associated with CP histological progression in patients without CP^17^. Thus, the previous and current results suggest that the histological condition of the pancreas may progress and lead to an increased number of microscopic precancerous lesions because of various factors such as age and smoking even in patients without CP. Patients with abnormal parenchymal EUS findings may need to be followed up more carefully than those without abnormal findings.

We further discovered that the number of PDGs, unlike PanINs and ADMs, was not significantly associated with pancreatic parenchymal EUS findings. These results may be because PanINs and ADMs significantly increased in response to CP histological conditions, but PDGs were weakly associated with CP histological conditions. PDG is reported to be a possible origin of IPMN^9^; however, chronic inflammation increases the risk of developing PanIN and PDAC^10^ but not IPMNs. Therefore, our results would be reasonable. In addition, there are only a few reports on PDGs in humans, and the significance of PDGs as precancerous lesions and their association with EUS findings and pathological features in the pancreatic parenchyma is not fully established. Thus, the unique aspect of this study was to indicate the relationship between PDGs and histological conditions in the pancreatic parenchyma using human specimens.

Our study had a few limitations. The most significant limitation is that none of the patients included in this study developed PDAC after pancreatic surgery; the clinical significance of predicting an increased number of microscopic precancerous lesions remains unclear, although abnormal parenchymal EUS findings were indeed associated with increased microscopic precancerous lesions. To clarify its clinical significance, accumulating cases of remnant pancreatic cancer and examining the relationship between EUS findings and precancerous lesions in the background pancreas at the time of the initial surgery is required in future studies. Second, our analyses utilized resected specimens of various types of pancreatobiliary diseases. The type of underlying tumors may affect the results, although there were no significant differences between each type of underlying tumor. Third, it is challenging to completely match the evaluation areas in the pancreas due to the retrospective nature of the study and the lack of clear landmarks. However, histological changes were widely observed across the sections and the differences between closely adjacent sections were minimal. These findings suggest that minor discrepancies in the evaluation sites are unlikely to significantly affect our overall results. However, the parenchymal EUS abnormalities and histological changes are sometimes localized, rather than diffuse involving the entire, pancreatic parenchyma. There may still be variations and heterogeneity within the pancreas that may not be captured by a single-slide analysis.

In conclusion, EUS findings in the pancreatic parenchyma were associated with the frequency of occurrence of microscopic precancerous lesions, especially PanINs and ADMs. Abnormal parenchymal EUS findings may help predict increased microscopic precancerous lesions, leading to risk stratification of developing PDAC.

## Supplementary Information

Supplementary Information 1.

Supplementary Information 2.

## Data Availability

All data generated during this study are included in this article and its Supplementary Information files.
