# Supplementary material for: Benefits of pancreatic parenchymal endoscopic ultrasonography in predicting microscopic precancerous lesions of pancreatic cancer
Source: Sci Rep. 2023 Jul 25;13:12052. doi: 10.1038/s41598-023-38920-1 (PMC10368726; doi:10.1038/s41598-023-38920-1)
Supplement: Supplementary file 1 — Supplementary Information 1. [file 41598_2023_38920_MOESM1_ESM.pdf]

Supplementary Figure S1

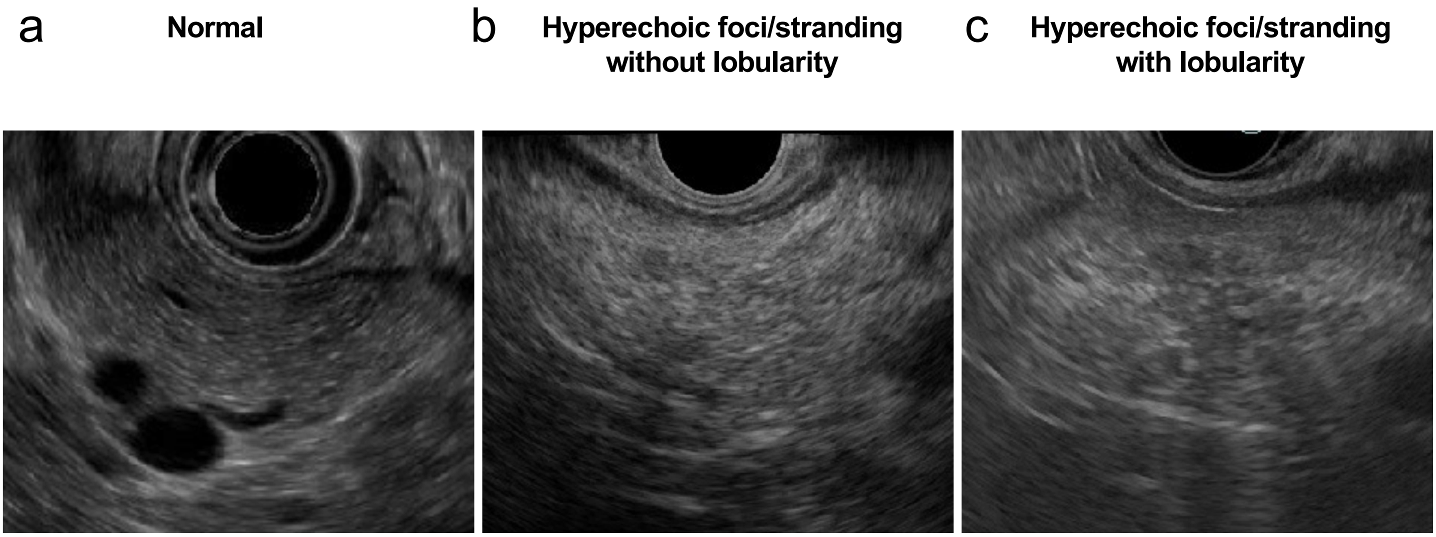

Supplementary Figure S1. A representative endoscopic ultrasonography imaging according to the Rosemont classification. **(a)** Normal findings. **(b)** Hyperechoic foci/stranding without lobularity. **(c)** Hyperechoic foci/stranding with lobularity.

Supplementary Figure S2

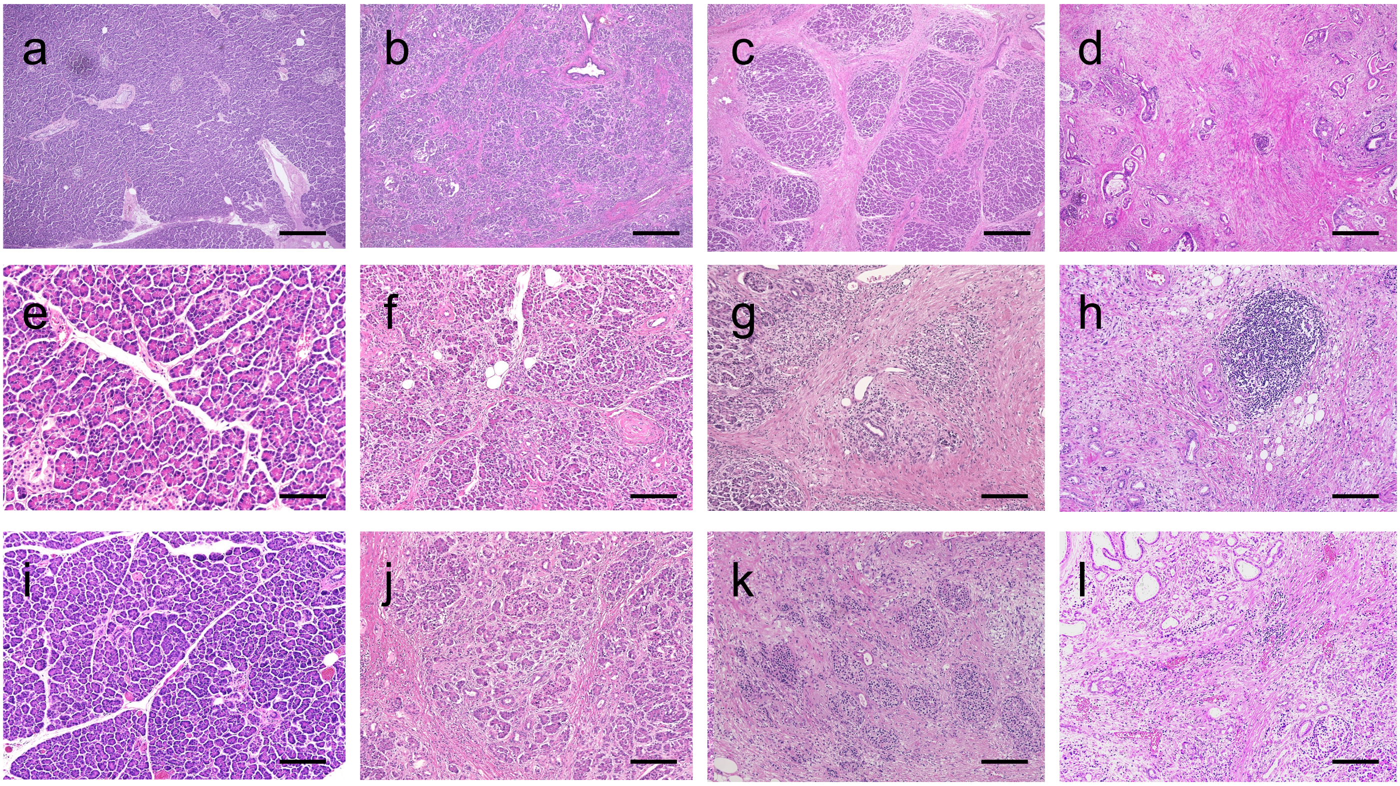

Supplementary Figure S2. Grading of chronic pancreatitis histological conditions. Images **a–d**, **e–h**, and **i–l** represent the grade of pancreatic fibrosis, inflammation, and atrophy, respectively. Scale bar in **a-d**, 500 µm, and in **e-l**, 100 µm.
(a, e, i) Grades 0 (none), **(b, f, j)** 1 (mild), **(c, g, k)** 2 (moderate), and **(d, h, l)** 3 (severe).

Supplementary Figure S3

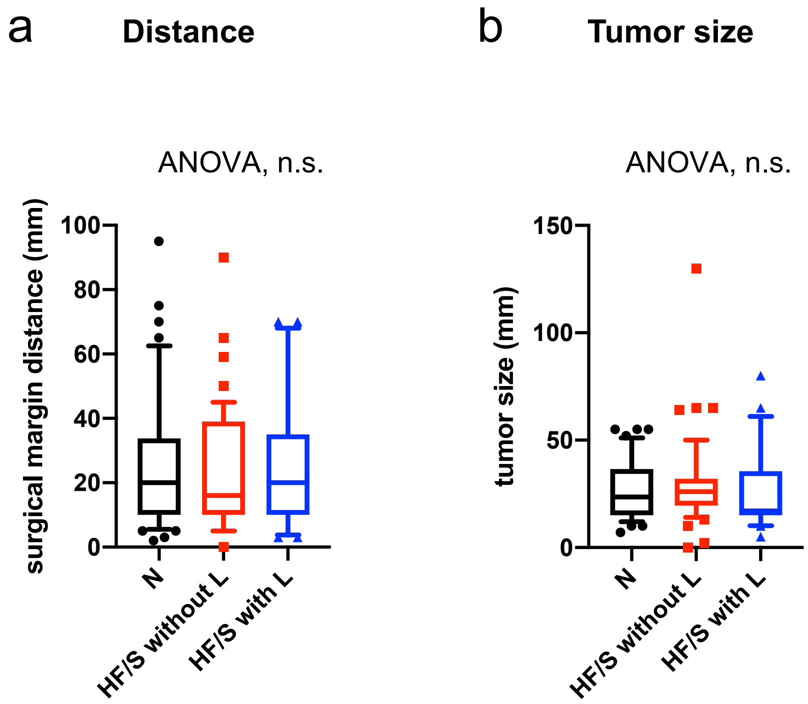

Supplementary Figure S3. The relationship between surgical margin distance and EUS findings **(a)** and between tumor size and EUS findings **(b)**. Error bars in **(a, b)**, mean ± standard deviation; n.s., not significant; N, normal; HF/S, hyperechoic foci/stranding; L, lobularity; by one-way analysis of variance

Supplementary Figure S4

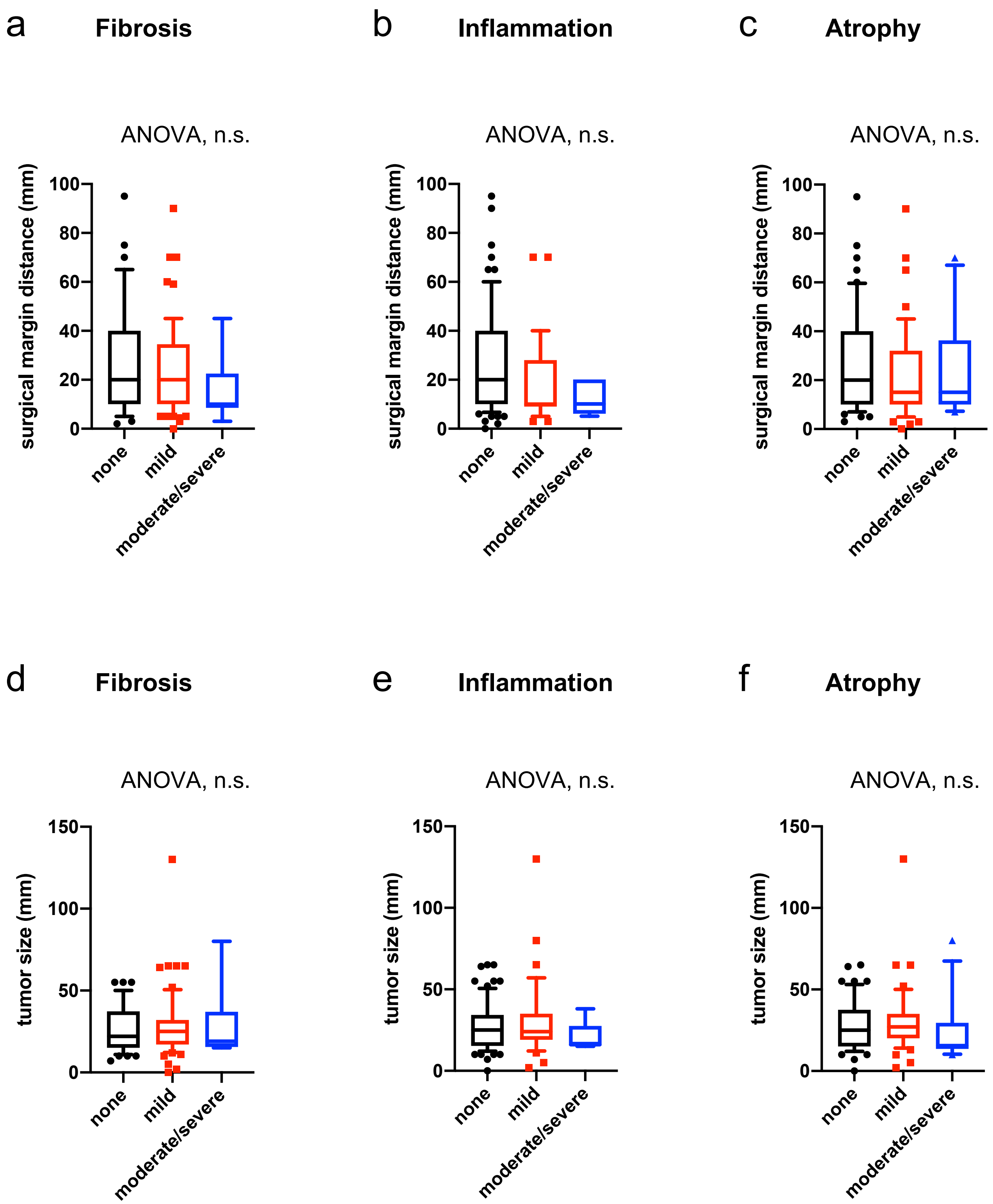

Supplementary Figure S4. The relationship between surgical margin distance and pathological findings, such as fibrosis **(a)**, inflammation **(b)**, and atrophy **(c)**. Error bars in **(a–f)**, mean ± standard deviation; n.s., not significant; N, normal; HF/S, hyperechoic foci/stranding; L, lobularity; by one-way analysis of variance

Supplementary Figure S5

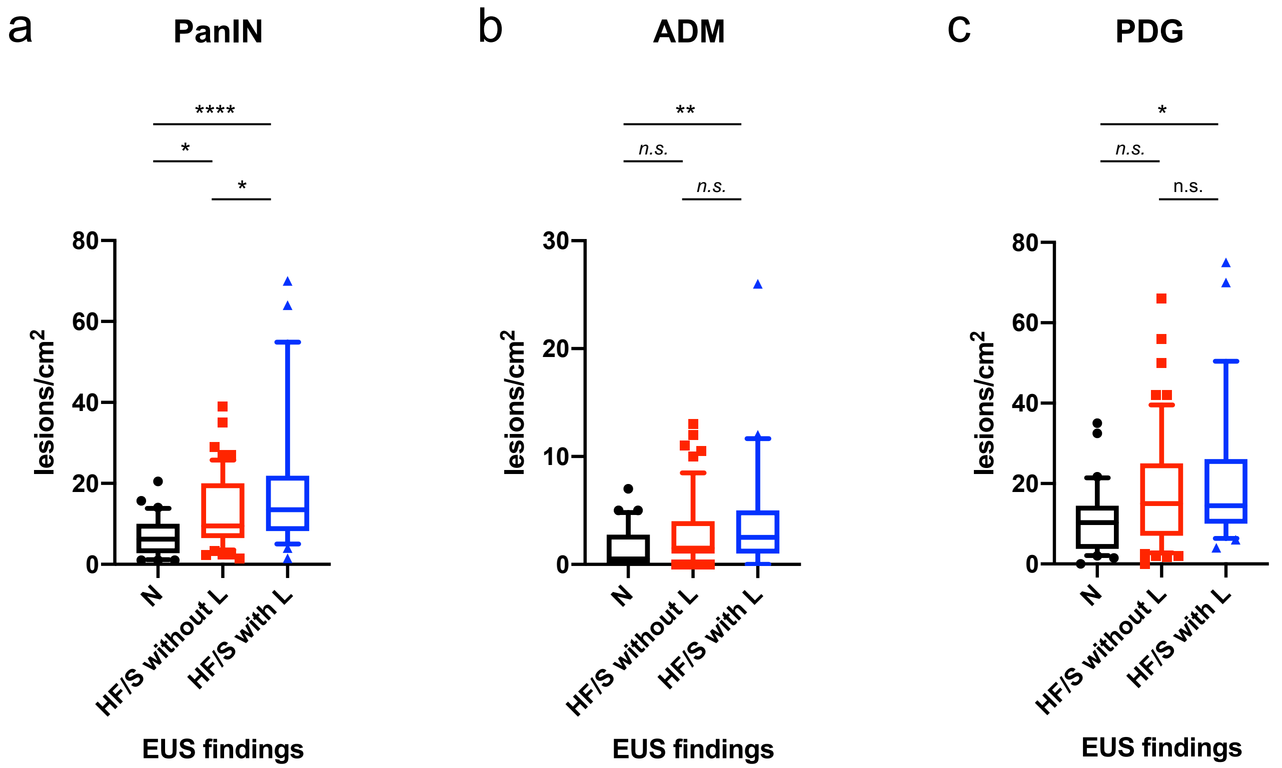

Supplementary Figure S5. The association between the frequency of microscopic precancerous lesions, such as pancreatic intraepithelial neoplasia **(a)**, acinar-to-ductal metaplasia **(b)**, and pancreatic duct gland **(c)**, and each endoscopic ultrasonography finding in the pancreatic parenchyma. Error bars in **(a–c)**, mean ± standard deviation; *$P < 0.05$, **$P < 0.01$, ***$P < 0.001$, ****$p < 0.0001$; n.s., not significant; N, normal; HF/S, hyperechoic foci/stranding; L, lobularity; by one-way analysis of variance with Tukey's test.
