# Supplementary material for: Benefits of pancreatic parenchymal endoscopic ultrasonography in predicting microscopic precancerous lesions of pancreatic cancer
Source: Sci Rep. 2023 Jul 25;13:12052. doi: 10.1038/s41598-023-38920-1 (PMC10368726; doi:10.1038/s41598-023-38920-1)
Supplement: Supplementary file 2 — Supplementary Information 2. [file 41598_2023_38920_MOESM2_ESM.pdf]

Benefits of pancreatic parenchymal endoscopic ultrasonography in predicting microscopic precancerous lesions of pancreatic cancer

Kohei Yamakawa[1#], Noriko Inomata[1#], Atsuhiro Masuda[1*], Mamoru Takenaka[2], Hirochika Toyama[3], Keitaro Sofue[4], Arata Sakai[1], Takashi Kobayashi[1], Takeshi Tanaka[1], Masahiro Tsujimae[1], Shigeto Ashina[1], Masanori Gonda[1], Shohei Abe[1], Shigeto Masuda[1], Hisahiro Uemura[1], Shinya Kohashi[1], Kae Nagao[1], Yoshiyuki Harada[1], Mika Miki[1], Yosuke Irie[1], Noriko Juri[1], Hideyuki Shiomi[5], Maki Kanzawa[6], Tomoo Itoh[6], Takumi Fukumoto[3], and Yuzo Kodama[1]

[1]Division of Gastroenterology, Department of Internal Medicine, Kobe University Graduate School of Medicine. [2]Department of Gastroenterology and Hepatology, Kindai University Faculty of Medicine. [3]Division of Hepato-Biliary-Pancreatic Surgery, Department of Surgery, Kobe University Graduate School of Medicine. [4]Department of Radiology, Kobe University Graduate School of Medicine. [5]Department of Internal Medicine, Division of Gastroenterology and Hepatology, Hyogo College of Medicine. [6]Division of Diagnostic Pathology, Kobe University Graduate School of Medicine. [#]These two authors contributed equally to this work.

Supplementary Table S1. Correlation of each pancreatic parenchymal endoscopic ultrasonography finding with histological features

EUS finding	No. of patients	No. of patients with fibrosis (%)	Fibrosis (outcome variable)	
			Univariate OR (95% CI)	Multivariate OR# (95% CI)
Normal	33	6 (18.2%)	1 (reference)	1 (reference)
Hyperechoic foci/stranding Without lobularity	55	40 (72.7%)	12.0 (4.1-34.8)	11.0 (3.6-33.3)
With lobularity	26	24 (92.3%)	54.0 (9.9-293.3)	65.7 (10.6-406.8)
P_{trend}*			< 0.001	< 0.001

EUS finding	No. of patients	No. of patients with inflammation (%)	Inflammation (outcome variable)	
			Univariate OR (95% CI)	Multivariate OR# (95% CI)
Normal	33	1 (3.0%)	1 (reference)	1 (reference)
Hyperechoic foci/stranding Without lobularity	55	20 (36.4%)	18.3 (2.3-144.2)	20.3 (2.5-165.8)
With lobularity	26	17 (65.4%)	60.4 (7.1-518.0)	112.6 (11.5-1103.2)
P_{trend}*			< 0.001	< 0.001

EUS finding	No. of patients	No. of patients with atrophy (%)	Atrophy (outcome variable)	
			Univariate OR (95% CI)	Multivariate OR# (95% CI)
Normal	33	5 (15.2%)	1 (reference)	1 (reference)
Hyperechoic foci/stranding Without lobularity	55	32 (58.2%)	7.8 (2.6-23.2)	7.6 (2.4-23.9)
With lobularity	26	24 (92.3%)	67.2 (11.9-378.3)	59.6 (10.2-348.6)
P_{trend}*			< 0.001	< 0.001

* P_{trend} is calculated via ordinal logistic regression analysis across the ordinal categories (normal, hyperechoic without lobularity, and lobularity) of pancreatic parenchymal EUS findings.

# The odds ratio (OR) is adjusted for age, smoking status, alcohol consumption, and the underlying tumor necessitating pancreatic surgery.

EUS, endoscopic ultrasonography; CI, confidence interval

Supplementary Table S2. Association of inflammation in the pancreatic parenchyma with the frequency of occurrence of microscopic precancerous lesions

**Histological grade of Inflammation**	**No. of cases with "high" frequency of PanIN**	**PanIN mean ± SD (lesion/cm²)**	**PanIN (outcome variable)**	
			Univariate OR (95% CI)	Multivariate OR# (95% CI)
Absence (none)	27 (37.3%)	9.1 ± 7.3	1 (reference)	1 (reference)
Presence (mild/moderate/severe)	29 (76.3%)	20.6 ± 15.5	5.8 (2.4-14.1)	5.8 (2.3-14.9)
P-value			< 0.001	< 0.001
	**No. of cases with "high" frequency of ADM**	**ADM mean ± SD (lesion/cm²)**	**ADM (outcome variable)**	
			Univariate OR (95% CI)	Multivariate OR# (95% CI)
Absence (none)	31 (40.8%)	2.2 ± 3.8	1 (reference)	1 (reference)
Presence (mild/moderate/severe)	30 (78.9%)	3.7 ± 3.3	5.4 (2.2-13.4)	3.3 (1.4-8.0)
P-value			< 0.001	0.007
	**No. of cases with "high" frequency of PDG**	**PDG mean ± SD (lesion/cm²)**	**PDG (outcome variable)**	
			Univariate OR (95% CI)	Multivariate OR# (95% CI)
Absence (none)	28 (37.3%)	12.9 ± 11.3	1 (reference)	1 (reference)
Presence (mild/moderate/severe)	28 (73.7%)	24.5 ± 17.2	4.8 (2.0-11.3)	4.4 (1.8-10.8)
P-value			< 0.001	0.001

The odds ratio (OR) was adjusted for age, smoking status, alcohol consumption, and the underlying tumor necessitating pancreatic surgery.

EUS, endoscopic ultrasonography; SD, standard deviation; PanIN, pancreatic intraepithelial neoplasm; ADM, acinar-to-ductal metaplasia; PDG, pancreatic duct gland; OR, odds ratio; CI, confidence interval

Supplementary Table S3. Association of atrophy in the pancreatic parenchyma with the frequency of occurrence of microscopic precancerous lesions

**Histological grade of Atrophy**	**No. of cases with "high" frequency of PanIN**	**PanIN mean ± SD (lesion/cm^2)**	**PanIN (outcome variable)**	
			Univariate OR (95% CI)	Multivariate OR# (95% CI)
Absence (none)	16 (30.2%)	8.1 ± 6.9	1 (reference)	1 (reference)
Presence (mild/moderate/severe)	40 (65.6%)	17.2 ± 13.8	4.4 (2.0-9.7)	6.7 (2.6-17.3)
P-value			< 0.001	< 0.001
**Histological grade of Atrophy**	**No. of cases with "high" frequency of ADM**	**ADM mean ± SD (lesion/cm^2)**	**ADM (outcome variable)**	
			Univariate OR (95% CI)	Multivariate OR# (95% CI)
Absence (none)	17 (32.1%)	2.0 ± 4.0	1 (reference)	1 (reference)
Presence (mild/moderate/severe)	44 (72.1%)	3.3 ± 3.3	5.5 (2.5-12.2)	5.2 (2.0-13.1)
P-value			< 0.001	< 0.001
**Histological grade of Atrophy**	**No. of cases with "high" frequency of PDG**	**PDG mean ± SD (lesion/cm^2)**	**PDG (outcome variable)**	
			Univariate OR (95% CI)	Multivariate OR# (95% CI)
Absence (none)	19 (35.8%)	12.1 ± 10.7	1 (reference)	1 (reference)
Presence (mild/moderate/severe)	37 (60.7%)	20.8 ± 16.2	2.8 (1.3-5.9)	3.0 (1.3-7.1)
P-value			0.008	0.01

The odds ratio (OR) was adjusted for age, smoking status, alcohol consumption, and the underlying tumor necessitating pancreatic surgery.

EUS, endoscopic ultrasonography; SD, standard deviation; PanIN, pancreatic intraepithelial neoplasm; ADM, acinar-to-ductal metaplasia; PDG, pancreatic duct gland; OR, odds ratio; CI, confidence interval
